# 2-(1,3-Benzoxazol-2-yl)guanidinium chloride

**DOI:** 10.1107/S1600536811044655

**Published:** 2011-10-29

**Authors:** Shaaban K. Mohamed, Peter N. Horton, Mahmoud A.A. El-Remaily, Seik Weng Ng

**Affiliations:** a Chemistry and Environmental Division, Manchester Metropolitan University, Manchester M15 6BH, England; b School of Chemistry, University of Southampton, Southampton SO17 1BJ, England; c Department of Chemistry, Faculty of Science, Sohag University, Egypt; d Department of Chemistry, University of Malaya, 50603 Kuala Lumpur, Malaysia; e Chemistry Department, King Abdulaziz University, PO Box 80203 Jeddah, Saudi Arabia

## Abstract

The non-H atoms of the cation of the title salt, C_8_H_9_N_4_O^+^·Cl^−^, are approximately co-planar (r.m.s. deviation = 0.024 Å) with one amino group forming an intra­molecular hydrogen bond to the tertiary N atom of the benzoxazole fused-ring system. The cations and anions are linked by cyclic *R*_2_^1^(6) N—H⋯Cl hydrogen-bonding associations, generating linear chains running along the *a*-axis direction.

## Related literature

For the synthesis, see: Takahashi & Niino (1943 ▶). For the structure of a co-crystal of 2-(1,3-benzoxazol-2-yl)guanidine, see: Bishop *et al.* (2005 ▶) and for the structure of 2-(1,3-benzothia­zol-2-yl)guanidine, see: Mohamed *et al.* (2011 ▶). For graph-set analysis, see: Etter *et al.* (1990 ▶).
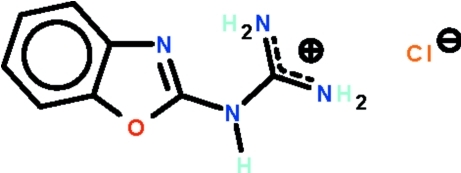


## Experimental

### 

#### Crystal data

C_8_H_9_N_4_O^+^·Cl^−^
*M* *_r_* = 212.64
Triclinic, 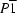

*a* = 6.669 (2) Å
*b* = 8.152 (4) Å
*c* = 9.630 (3) Å
α = 65.062 (2)°
β = 85.020 (2)°
γ = 73.710 (2)°
*V* = 455.4 (3) Å^3^
*Z* = 2
Mo *K*α radiation
μ = 0.39 mm^−1^
*T* = 120 K
0.20 × 0.05 × 0.04 mm

#### Data collection

Bruker–Nonius Roper CCD camera on κ-goniostat diffractometer
Absorption correction: multi-scan (*SADABS*; Sheldrick, 1996 ▶) *T*_min_ = 0.926, *T*_max_ = 0.985
8184 measured reflections
2088 independent reflections
1895 reflections with *I* > 2σ(*I*)
*R*_int_ = 0.044

#### Refinement

*R*[*F* ^2^ > 2σ(*F* ^2^)] = 0.036
*wR*(*F* ^2^) = 0.089
*S* = 1.03
2088 reflections
147 parameters
5 restraints
H atoms treated by a mixture of independent and constrained refinement
Δρ_max_ = 0.31 e Å^−3^
Δρ_min_ = −0.26 e Å^−3^

### 

Data collection: *COLLECT* (Hooft, 1998 ▶); cell refinement: *DENZO* (Otwinowski & Minor, 1997 ▶) and *COLLECT*; data reduction: *DENZO*; program(s) used to solve structure: *SHELXS97* (Sheldrick, 2008 ▶); program(s) used to refine structure: *SHELXL97* (Sheldrick, 2008 ▶); molecular graphics: *X-SEED* (Barbour, 2001 ▶); software used to prepare material for publication: *publCIF* (Westrip, 2010 ▶).

## Supplementary Material

Crystal structure: contains datablock(s) global, I. DOI: 10.1107/S1600536811044655/zs2158sup1.cif

Structure factors: contains datablock(s) I. DOI: 10.1107/S1600536811044655/zs2158Isup2.hkl

Supplementary material file. DOI: 10.1107/S1600536811044655/zs2158Isup3.cml

Additional supplementary materials:  crystallographic information; 3D view; checkCIF report

## Figures and Tables

**Table 1:** Hydrogen-bond geometry (Å, °)

*D*—H⋯*A*	*D*—H	H⋯*A*	*D*⋯*A*	*D*—H⋯*A*
N2—H1⋯Cl1	0.88 (1)	2.18 (1)	3.047 (2)	168 (2)
N3—H2⋯Cl1	0.88 (1)	2.67 (2)	3.415 (2)	144 (2)
N3—H3⋯Cl1^i^	0.87 (1)	2.53 (2)	3.297 (2)	147 (2)
N4—H4⋯Cl1^i^	0.88 (1)	2.33 (1)	3.168 (2)	160 (2)
N4—H5⋯N1	0.88 (1)	2.08 (2)	2.765 (2)	134 (2)

Symmetry code: (i) 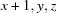.

**Fig. 1.**
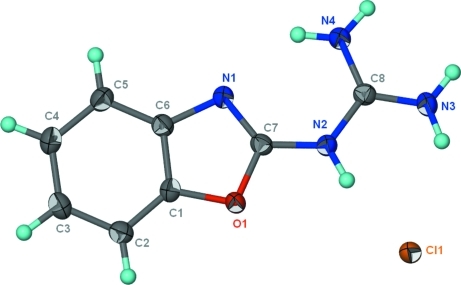
Thermal ellipsoid plot (Barbour, 2001) of C8H9N4O+ Cl- at the 70% probability level. Hydrogen atoms are drawn as spheres of arbitrary radius.

## supplementary crystallographic information

### Comment

The preceding study reports 2-(1,3-benzothiazol-2-yl)guanidinium chloride (Mohamed *et al.*, 2011). Replacing the sulfur in the fused-ring by oxygen leads to the analogous compound, 2-(1,3-benzoxazol-2-yl)guandinium chloride (Scheme I). However, this salt and 2-(1,3-benzothiazol-2-yl)guanidinium chloride are are not isostructural as they belong to different crystal systems. The non-H atoms of the cation of the title salt, C_8_H_9_N_4_O^+^ Cl^-^ (Fig. 1), lie on a plane with one amino group forming an intramolecular hydrogen bond to the tertiary N atom of the benzoxazole fused-ring.  The cations and anions are linked by cyclic *R*^1^_2_(6) N—H···Cl hydrogen-bonding associations [Etter *et al.*, 1990)], to generate linear chains running along the *a*-axis of the tricinic unit cell (Table 1). This salt was first reported in 1943 (Takahashi & Niino, 1943). The structure of a co-crystal of 2-(1,3-benzoxazol-2-yl)guanidine has been reported (Bishop *et al.*, 2005).

### Experimental

2-(1,3-Benzoxazol-2-yl)guanidine was synthesized by using a literature procedure similar to that used for synthesizing 2-(1,3-benzothioazol-2-yl)guanidine (Takahashi & Niino, 1943). The guanidine (0.05 mol) was heated in ethanol (50 ml) in the presence of a few drops of hydrochloric acid for 3 h. The mixture was cooled and the product was recrystallized from ethanol to give the title compound (m.p. 538 K)in 95% yield.

### Refinement

Carbon-bound H-atoms were placed in calculated positions (C—H 0.95 Å) and were included in the refinement in the riding model approximation, with *U*_iso_(H) set to 1.2*U*_eq_(C). The amino H-atoms were located in a difference Fourier map, and were refined with a distance restraint of N–H = 0.88±0.01 Å, with their isotropic displacement parameters freely refining.

### Crystal data

**Table:**

C_8_H_9_N_4_O^+^·Cl^−^	*Z* = 2
*M**_r_* = 212.64	*F*(000) = 220
Triclinic, *P*1	*D*_x_ = 1.551 Mg m^−^^3^
Hall symbol: -P 1	Melting point: 538 K
*a* = 6.669 (2) Å	Mo *K*α radiation, λ = 0.71073 Å
*b* = 8.152 (4) Å	Cell parameters from 1979 reflections
*c* = 9.630 (3) Å	θ = 2.9–27.5°
α = 65.062 (2)°	µ = 0.39 mm^−^^1^
β = 85.020 (2)°	*T* = 120 K
γ = 73.710 (2)°	Prism, colorless
*V* = 455.4 (3) Å^3^	0.20 × 0.05 × 0.04 mm

### Data collection

**Table:**

Bruker–Nonius Roper CCD camera on κ-goniostat diffractometer	2088 independent reflections
Radiation source: Bruker-Nonius FR591 rotating anode	1895 reflections with *I* > 2σ(*I*)
graphite	*R*_int_ = 0.044
Detector resolution: 9.091 pixels mm^-1^	θ_max_ = 27.5°, θ_min_ = 3.2°
φ and ω scans	*h* = −8→8
Absorption correction: multi-scan (*SADABS*; Sheldrick, 1996)	*k* = −10→10
*T*_min_ = 0.926, *T*_max_ = 0.985	*l* = −12→12
8184 measured reflections	

### Refinement

**Table:**

Refinement on *F*^2^	Primary atom site location: structure-invariant direct methods
Least-squares matrix: full	Secondary atom site location: difference Fourier map
*R*[*F*^2^ > 2σ(*F*^2^)] = 0.036	Hydrogen site location: inferred from neighbouring sites
*wR*(*F*^2^) = 0.089	H atoms treated by a mixture of independent and constrained refinement
*S* = 1.03	*w* = 1/[σ^2^(*F*_o_^2^) + (0.0256*P*)^2^ + 0.4591*P*] where *P* = (*F*_o_^2^ + 2*F*_c_^2^)/3
2088 reflections	(Δ/σ)_max_ = 0.001
147 parameters	Δρ_max_ = 0.31 e Å^−^^3^
5 restraints	Δρ_min_ = −0.26 e Å^−^^3^

### Special details

**Table:**

Geometry. All e.s.d.'s (except the e.s.d. in the dihedral angle between two l.s. planes) are estimated using the full covariance matrix. The cell e.s.d.'s are taken into account individually in the estimation of e.s.d.'s in distances, angles and torsion angles; correlations between e.s.d.'s in cell parameters are only used when they are defined by crystal symmetry. An approximate (isotropic) treatment of cell e.s.d.'s is used for estimating e.s.d.'s involving l.s. planes.

### Fractional atomic coordinates and isotropic or equivalent isotropic displacement parameters (Å^2^)

**Table:**

	*x*	*y*	*z*	*U*_iso_*/*U*_eq_	
Cl1	0.25918 (6)	0.37898 (6)	0.41313 (5)	0.01998 (13)	
O1	0.50373 (17)	−0.01606 (16)	0.27313 (13)	0.0176 (3)	
N1	0.8440 (2)	−0.0414 (2)	0.21101 (16)	0.0179 (3)	
N2	0.6679 (2)	0.1505 (2)	0.34140 (17)	0.0186 (3)	
N3	0.7839 (2)	0.3256 (2)	0.43342 (18)	0.0215 (3)	
N4	1.0159 (2)	0.1588 (2)	0.31608 (18)	0.0212 (3)	
C1	0.5575 (3)	−0.1365 (2)	0.19970 (19)	0.0171 (3)	
C2	0.4318 (3)	−0.2237 (2)	0.1669 (2)	0.0197 (3)	
H2A	0.2904	−0.2110	0.1960	0.024*	
C3	0.5268 (3)	−0.3327 (2)	0.0875 (2)	0.0224 (4)	
H3A	0.4482	−0.3975	0.0619	0.027*	
C4	0.7347 (3)	−0.3490 (2)	0.0448 (2)	0.0221 (4)	
H4A	0.7930	−0.4229	−0.0106	0.027*	
C5	0.8592 (3)	−0.2595 (2)	0.0815 (2)	0.0210 (4)	
H5A	1.0010	−0.2719	0.0533	0.025*	
C6	0.7658 (3)	−0.1517 (2)	0.16100 (19)	0.0178 (3)	
C7	0.6845 (2)	0.0303 (2)	0.27334 (19)	0.0171 (3)	
C8	0.8270 (2)	0.2129 (2)	0.36229 (19)	0.0169 (3)	
H1	0.544 (2)	0.201 (3)	0.367 (3)	0.035 (6)*	
H2	0.654 (2)	0.367 (4)	0.454 (3)	0.046 (7)*	
H3	0.881 (3)	0.372 (3)	0.446 (3)	0.039 (7)*	
H4	1.110 (3)	0.205 (3)	0.334 (2)	0.025 (5)*	
H5	1.035 (4)	0.085 (3)	0.268 (2)	0.035 (6)*	

### Atomic displacement parameters (Å^2^)

**Table:**

	*U*^11^	*U*^22^	*U*^33^	*U*^12^	*U*^13^	*U*^23^
Cl1	0.0149 (2)	0.0214 (2)	0.0280 (2)	−0.00545 (15)	0.00285 (15)	−0.01445 (17)
O1	0.0145 (5)	0.0191 (6)	0.0231 (6)	−0.0051 (4)	0.0008 (4)	−0.0122 (5)
N1	0.0169 (7)	0.0190 (7)	0.0208 (7)	−0.0048 (5)	0.0015 (5)	−0.0111 (6)
N2	0.0133 (7)	0.0216 (7)	0.0259 (8)	−0.0041 (5)	0.0015 (5)	−0.0151 (6)
N3	0.0182 (7)	0.0238 (8)	0.0295 (8)	−0.0066 (6)	0.0011 (6)	−0.0170 (7)
N4	0.0162 (7)	0.0250 (8)	0.0288 (8)	−0.0068 (6)	0.0028 (6)	−0.0169 (7)
C1	0.0188 (8)	0.0156 (8)	0.0171 (8)	−0.0032 (6)	−0.0001 (6)	−0.0076 (6)
C2	0.0180 (8)	0.0196 (8)	0.0215 (8)	−0.0062 (6)	0.0008 (6)	−0.0079 (7)
C3	0.0241 (9)	0.0211 (9)	0.0249 (9)	−0.0074 (7)	−0.0021 (7)	−0.0110 (7)
C4	0.0247 (9)	0.0208 (9)	0.0232 (9)	−0.0040 (7)	0.0007 (7)	−0.0127 (7)
C5	0.0195 (8)	0.0221 (9)	0.0225 (9)	−0.0039 (7)	0.0025 (7)	−0.0118 (7)
C6	0.0173 (8)	0.0176 (8)	0.0194 (8)	−0.0049 (6)	−0.0002 (6)	−0.0081 (7)
C7	0.0147 (7)	0.0175 (8)	0.0199 (8)	−0.0046 (6)	−0.0009 (6)	−0.0079 (7)
C8	0.0153 (7)	0.0170 (8)	0.0176 (8)	−0.0037 (6)	−0.0005 (6)	−0.0067 (7)

### Geometric parameters (Å, °)

**Table:**

O1—C7	1.361 (2)	N4—H5	0.879 (10)
O1—C1	1.391 (2)	C1—C2	1.371 (2)
N1—C7	1.292 (2)	C1—C6	1.391 (2)
N1—C6	1.409 (2)	C2—C3	1.395 (3)
N2—C8	1.361 (2)	C2—H2A	0.9500
N2—C7	1.368 (2)	C3—C4	1.397 (3)
N2—H1	0.879 (10)	C3—H3A	0.9500
N3—C8	1.321 (2)	C4—C5	1.398 (2)
N3—H2	0.875 (10)	C4—H4A	0.9500
N3—H3	0.874 (10)	C5—C6	1.387 (2)
N4—C8	1.318 (2)	C5—H5A	0.9500
N4—H4	0.876 (9)		
			
C7—O1—C1	102.84 (12)	C2—C3—H3A	119.2
C7—N1—C6	102.87 (14)	C4—C3—H3A	119.2
C8—N2—C7	125.13 (14)	C3—C4—C5	121.75 (16)
C8—N2—H1	115.8 (16)	C3—C4—H4A	119.1
C7—N2—H1	118.9 (16)	C5—C4—H4A	119.1
C8—N3—H2	119.2 (18)	C6—C5—C4	116.77 (16)
C8—N3—H3	119.4 (16)	C6—C5—H5A	121.6
H2—N3—H3	121 (2)	C4—C5—H5A	121.6
C8—N4—H4	115.4 (14)	C5—C6—C1	119.91 (16)
C8—N4—H5	118.0 (16)	C5—C6—N1	131.01 (16)
H4—N4—H5	127 (2)	C1—C6—N1	109.06 (14)
C2—C1—O1	127.63 (15)	N1—C7—O1	117.68 (15)
C2—C1—C6	124.81 (16)	N1—C7—N2	129.18 (15)
O1—C1—C6	107.55 (14)	O1—C7—N2	113.14 (14)
C1—C2—C3	115.05 (16)	N4—C8—N3	122.14 (16)
C1—C2—H2A	122.5	N4—C8—N2	120.88 (15)
C3—C2—H2A	122.5	N3—C8—N2	116.97 (15)
C2—C3—C4	121.70 (16)		
			
C7—O1—C1—C2	−179.20 (17)	O1—C1—C6—N1	0.35 (18)
C7—O1—C1—C6	−0.35 (17)	C7—N1—C6—C5	177.86 (18)
O1—C1—C2—C3	178.07 (15)	C7—N1—C6—C1	−0.19 (18)
C6—C1—C2—C3	−0.6 (3)	C6—N1—C7—O1	0.0 (2)
C1—C2—C3—C4	−0.4 (3)	C6—N1—C7—N2	−179.71 (17)
C2—C3—C4—C5	1.1 (3)	C1—O1—C7—N1	0.26 (19)
C3—C4—C5—C6	−0.7 (3)	C1—O1—C7—N2	179.97 (14)
C4—C5—C6—C1	−0.2 (3)	C8—N2—C7—N1	−3.1 (3)
C4—C5—C6—N1	−178.12 (17)	C8—N2—C7—O1	177.25 (15)
C2—C1—C6—C5	0.9 (3)	C7—N2—C8—N4	0.0 (3)
O1—C1—C6—C5	−177.95 (15)	C7—N2—C8—N3	−178.54 (16)
C2—C1—C6—N1	179.24 (16)		

### Hydrogen-bond geometry (Å, °)

**Table:**

*D*—H···*A*	*D*—H	H···*A*	*D*···*A*	*D*—H···*A*
N2—H1···Cl1	0.88 (1)	2.18 (1)	3.047 (2)	168 (2)
N3—H2···Cl1	0.88 (1)	2.67 (2)	3.415 (2)	144 (2)
N3—H3···Cl1^i^	0.87 (1)	2.53 (2)	3.297 (2)	147 (2)
N4—H4···Cl1^i^	0.88 (1)	2.33 (1)	3.168 (2)	160 (2)
N4—H5···N1	0.88 (1)	2.08 (2)	2.765 (2)	134 (2)

Symmetry codes: (i) *x*+1, *y*, *z*.
